# Postoperative adjuvant treatment strategy for hepatocellular carcinoma with microvascular invasion: a non-randomized interventional clinical study

**DOI:** 10.1186/s12885-020-07087-7

**Published:** 2020-07-01

**Authors:** Liming Wang, Weihu Wang, Weiqi Rong, Zhuo Li, Fan Wu, Yunhe Liu, Yiling Zheng, Kai Zhang, Tana Siqin, Mei Liu, Bo Chen, Jianxiong Wu

**Affiliations:** 1 Department of Hepatobiliary Surgery, National Cancer Center/ National Clinical Research Center for Cancer/ Cancer Hospital, Chinese Academy of Medical Sciences and Peking Union Medical College, 17 Panjiayuan Nanli Area, Chaoyang District, Beijing, 100021 China; 2 Department of Radiation Oncology, Key laboratory of Carcinogenesis and Translational Research (Ministry of Education/Beijing), Peking University Cancer Hospital & Institute, 52 Fucheng Rd, Haidian District, Beijing, 100142 China; 3 Department of Pathology, National Cancer Center/ National Clinical Research Center for Cancer /Cancer Hospital, Chinese Academy of Medical Sciences and Peking Union Medical College, 17 Panjiayuan Nanli Area, Chaoyang District, Beijing, 100021 China; 4 Laboratory of Cell and Molecular Biology & State Key Laboratory of Molecular Oncology, National Cancer Center/ National Clinical Research Center for Cancer /Cancer Hospital, Chinese Academy of Medical Sciences and Peking Union Medical College, 17 Panjiayuan Nanli Area, Chaoyang District, Beijing, 100021 China; 5 Department of Radiation Oncology, National Cancer Center/ National Clinical Research Center for Cancer /Cancer Hospital, Chinese Academy of Medical Sciences and Peking Union Medical College, 17 Panjiayuan Nanli Area, Chaoyang District, Beijing, 100021 China

**Keywords:** Hepatocellular carcinoma, Microvascular invasion, Radiotherapy, Relapse survival, Overall survival

## Abstract

**Background:**

Microvascular invasion (MVI) is considered to be one of the important prognostic factors that affect postoperative recurrence in patients with hepatocellular carcinoma (HCC) with variable results across their treatment options. This study was carried out to investigate efficacy of postoperative adjuvant RT in HCC patients with MVI.

**Methods:**

This was single center, prospective study carried out in HCC patients with MVI, aged 35–72 years. All patients were non-randomly allocated to receive standard postoperative treatment of HBV/HCV and nutritional therapy or RT in addition to standard postoperative treatment (1:1). The primary endpoints assessed were relapse-free survival and overall survival. The prognostic factors associated with survival outcomes were also analyzed. The safety events were graded according to NCI-CTCAE v4.03 criteria.

**Results:**

Of the 115 patients eligible for study, 59 patients were included in analysis. Univariate analysis revealed that MVI classification (*P* = 0.009), post-operative treatment strategies (*P* = 0.009) were prognostic factors for worst RFS; tumor size (*P* = 0.011), MVI classification (*P* = 0.005) and post-operative treatment (*P* = 0.015) were associated for OS. The 1-, 2-, 3-year RFS rates were 86.2, 70.5 and 63.4% for patients in RT group, and 46.4, 36.1, and 36.1% in control group. For OS, corresponding rates were 96.6, 80.7, and 80.7% for patients in RT group and 79.7, 58.3, and 50.0% in control group. Subgroup classification of HCC patients according to low risk MVI showed significantly longer RFS (*P* = 0.035) and OS (*P* = 0.004) in RT group than control group, while for high risk MVI, RT depicted longer OS than control group with no significance (*P* = 0.106). Toxicities were usually observed in acute stage with no grade 4 toxicities.

**Conclusion:**

Postoperative adjuvant RT following hepatectomy offers better RFS for HCC patients with MVI than with standard postoperative therapy. Also, it will be useful to control microscopic lesions in both M1 (low risk) and M2 (high risk) subgroups of HCC patients with MVI.

**Trial registration:**

Trial Registration number: ChiCTR1800017371. Date of Registration: 2018-07-26. Registration Status: Retrospectively registered.

## Background

Hepatocellular carcinoma (HCC) is the seventh most common cancer and the second leading cause of cancer-related mortality in the world, with an estimated 841,080 new cancer cases and accounts for 781,631 deaths worldwide [1]. In China, HCC is the second most common cancer and accounts for 55% of all primary liver cancer cases [2]. Every year, approximately, 383,203 Chinese patients die of HCC which is responsible for 51% of liver cancer deaths worldwide [3]. Liver resection (LR) is one of the most efficient and curative treatment option for HCC patients [4]. However, < 30% of patients with HCC are eligible for surgery [5]. Also, one of the major complication post liver resection is the recurrence of HCC, reaching an incidence of more than 70% at 5 years [6], resulting in an unsatisfactory 5-year survival rate of less than 50% [7].

Several risk factors have been identified for recurrence of HCC including tumor size (> 2–3 cm), tumor number (2–3 nodule), peritumoral capsule, position of module near large vessels, partial necrosis, pattern of lipiodol accumulation, vascular invasion (both macroscopic and microscopic), presence of stellate nodules, histopathological grade, underlying cirrhosis and the type of surgery (i.e. narrow vs. wide surgical margins, anatomic vs. non-anatomic resection) [6, 8] Microvascular invasion (MVI), also known as intravascular cancer embolus, refers to the cancer cell nest in vessels lined with endothelial cells [9]. MVI may promote intrahepatic metastasis as the distance between the micrometastasis and the main tumor is ≤10 mm in most of the patients [10, 11]. In a recent systematic review, the prevalence of MVI in HCC patients ranged from 15 to 57.1% [9]. Presence of MVI has been reported to be one of the important risk factors associated with early postoperative recurrence within 2 years [12–16]. The recurrence-free survival rates at 3 years for HCC patients with or without MVI were 27.7 and 62.5% respectively, while at 2 years for patients without MVI, with mild MVI and severe MVI were 75.9, 47.2 and 32.7% respectively [17]. MVI is thus considered to be a prognostic factor associated with lower survival and higher recurrence rates [18]. Even for patients with small HCCs, MVI increase the recurrence of HCC and has an adverse impact on the long-term survival [19, 20]. However, MVI cannot be diagnosed preoperatively. Though presence of MVI is a risk factor for recurrence of HCC, it can be confirmed only by postoperative pathology examination [21, 22]. Thus, providing such patients with optimal effective postoperative treatment is crucial.

The postoperative adjuvant treatment options for HCC includes transcatheter arterial chemoembolization (TACE), radiotherapy (RT), sorafenib, interferon, polyprenoic acid, adoptive immunotherapy and iodine-131-labeled lipiodol to decrease the recurrence and prolong the survival. Technical advances in radiotherapy including intensity modulated radiotherapy (IMRT), stereotactic body radiation therapy (SBRT) and proton beam RT have improved the risk-benefit ratio as they are minimally invasive, deliver higher RT doses to tumor volumes and achieve comparable or more better outcomes than other forms of liver-directed therapy for localized and locally advanced HCC [23–25]. RT offer high local control rates in case of unresectable HCC, can provide a modality to help bridge patients to potentially curative resection or transplantation. IMRT is an advanced technique that uses modulated beams which allow for more improved target coverage, more conformal radiation dose distribution, and better radiation dose sparing of critical normal structures other than the liver. The effectiveness of postoperative adjuvant RT in reducing the recurrence and improving the OS has been well documented in various studies [26–30]. Moreover, adjuvant RT following hepatectomy could efficiently improve the relapse free survival (RFS) and overall survival (OS) in HCC patients with MVI compared with TACE [21, 22]. However, real-world studies comparing the optimal postoperative adjuvant treatment for preventing recurrence of HCC in patients with MVI are limited.

In order to address the issue, the present study aimed to evaluate the long-term survival outcomes for HCC patients with MVI who received RT as their postoperative adjuvant treatment after curative hepatectomy. We hypothesized that adjuvant radiotherapy can be an effective treatment that might modulate the deleterious postoperative result. The prognostic factors associated with survival outcomes were also analyzed. This is the first clinical study to detect RT as the postoperative adjuvant treatment for HCC patients with MVI on their long-term survival.

## Methods

### Study design

This was a non-randomized, interventional study where data was collected retrospectively for all eligible HCC patients with MVI and prior curative hepatectomy from July 2015 to December 2018. The patients were assigned to either intervention or control group in ratio of 1:1. The postoperative adjuvant treatment was either radiotherapy or control and the choice to treatment was determined by the clinical experience of physicians and by the patient preference. The control group underwent standard postoperative treatment such as anti-viral (HBV/HCV) and nutritional therapy while the intervention group underwent a course of postoperative RT in addition to the standard postoperative treatment. The primary end point was RFS; the secondary end point was OS.

The study protocol was approved by Ethics Committee (Institutional Review Board) of Cancer Hospital, Chinese Academy of Medical Sciences (NCC2015 YZ-25) and conducted in accordance with the Declaration of Helsinki. All patients provided written informed consent before participation in the study. The trial was retrospectively registered at www.chictr.org.cn (ChiCTR1800017371).

### Eligibility criteria

The patients were included based on the following criteria’s: (1) male and female aged < 75 years; (2) primary HCC treated with curative surgical liver resection; (3) surgical margin less than 10 mm but microscopically free of tumor, (4) No presence of macro-vascular invasion but MVI were proven by postoperative pathology; (5) not more than two lesions, double primary tumor proven by postoperative pathology without intra or extrahepatic metastasis; (6) no tumor fracture and hemorrhage before and during resection; (7) Preoperative liver function was Child-Pugh A degree and Postoperative liver function recovered to Child-Pugh A degree in 4 weeks; (8) previous hepatitis B virus (HBV) infection confirmed by serological detection; (9) No severe cardiopulmonary or metabolic system dysfunction. The patients were excluded if they showed the presence of any one of the following: (1) postoperative intra or extrahepatic metastases within 4 weeks (2) postoperative liver failure or severe complications/adverse events within 4 weeks; (3) had simultaneous malignant tumor/diseases; (4) RT was performed as preoperative or intraoperative adjuvant treatment; (5) TACE was performed as postoperative adjuvant treatment; (6) sensitivity to radiation therapy.

### Sample size estimation

Based on the previous findings, postoperative RT can effectively control local micro-metastases in HCC patients with microvascular invasion. According to the principle of the difference test formula, it was assumed that the 2-year recurrence-free survival rate would reach 70% vs. 30% in the treatment group and the control group, with a two tailed α value of 0.05, and the test efficiency (power, i.e. 1-β) of 0.9. The dropout rate was assumed to be 10% and the ratio of cases between the treatment group and the control group was 1:1 where the patient was non-randomly enrolled to the control or the intervention group; the enrollment period was for 2 years, the enrollment period was 2 years, and the frequency was evenly divided. Each patient was followed up for at least 1 year. Power Analysis and Sample Size (PASS) software was used to estimate the sample size based on the previous trials [21, 22].

### Pathological diagnosis and MVI classification

The pathological diagnosis and classification of MVI were done according to the 2015 clinicopathological evidence-based practice guidelines for standardized pathological diagnosis of primary liver cancers in China. The classification of MVI is defined as follows: M0: no MVI; M1: low risk (the number of MVI is < 5 and at a distance of ≤1 cm from the tumor capsule); M2: high risk (the number of MVI is > 5 or at a distance of > 1 cm from the tumor capsule) [31].

### Postoperative treatment regimen

Nutritional and anti-HBV therapy were given to all the patients continuously as basic therapy to improve liver function, block the process of liver cirrhosis and prevent recurrence.

RT procedure was performed with intensity modulated radiation therapy (IMRT) that was generated for each patient in RT group. For patients in RT group, computed tomography scan was performed with the patients in a supine position, along with thermoplastic mask immobilization. The 4D-CT simulations were performed for all RT patients after 2017. The clinical target volume (CTV) was defined as the tumor cutting bed, indicated by postoperative CT/MR, with a 1-cm margin in three dimensions. If 4D-CT is unavailable, a margin of 1.0 cm was added in cranial-caudal directions and 0.5 cm in other directions to generate the planning target volume (PTV) by expanding CTV. For patients with images of 4D-CT simulations, PTV was generated according to motion of 4D-CT. The prescription dose for 95% PTV was 54–60 Gy which was delivered using 2 Gy per fraction for 5 days (fraction) per week. Details regarding the dose–volume constraints were as follows: mean dose for the whole liver was ≤24 Gy; maximum dose for stomach, duodenum, colon and spinal cord were ≤ 54 Gy, ≤ 54 Gy, ≤ 55 Gy and ≤ 40 Gy; V20 of left and right kidney was ≤30% respectively. The plans were generated and optimized independently and reviewed by 2 physicians and a physicist. All patients received image-guided radiotherapy using linear accelerators equipped with kilovolt cone beam CT (CBCT). CBCT was applied to patients for the first five fractions and then once a week if the setup error were less than 0.5 cm.

### Follow up

Similar to the previous studies [21, 22], all patients were followed-up quarterly following discharge from the hospital. Follow-up tests such as alpha-fetoprotein (AFP), liver function, chest X-ray, enhanced magnetic resonance imaging (MRI) and/or enhanced computed tomography (CT) were performed on patients. The patients were diagnosis of recurrence based on typical imaging findings and/or continually increased serum AFP. Further, biopsy was conducted to assess the histopathology or cytopathology evidence but not essentially for the assessment of recurrences.

RFS was defined as the time interval between the surgery date and the date of the first detection of recurrence or censored on the date of the last follow-up. OS was recorded as time period from the date of surgery to death or censored on the date of the last follow-up. The last follow-up was carried out in March 2019.

### Treatment for recurrence

The treatment strategy for recurrence of HCC was determined based on the comprehensive consideration of tumor characteristics, liver function and general condition by a multidisciplinary team. Local or regional curative treatment consisting of reoperation-hepatectomy, radiofrequency ablation (RFA) and stereotactic body radiation therapy (SBRT) was undertaken for nodular recurrence. Systemic palliative treatment such as TACE, molecular targeted therapy and chemotherapy were performed as alternative methods for diffuse recurrence.

### Safety

Toxicity was evaluated according to the National Cancer Institute Common Terminology Criteria for Adverse Events (NCI-CTCAE), version 3.0. Hematologic adverse effects were graded using the Radiation Therapy Oncology Group Morbidity Scoring Criteria [32]. Radiation-induced liver disease was defined as a minimum of 2-fold increase in anicteric elevation of alkaline phosphatase (ALP) levels and nonmalignant ascites, or a minimum 5-fold increase in transaminase levels above the normal upper limit or relative to pretreatment levels. Acute toxicity was defined as events occurring during treatment or within the first month after treatment. Late toxicity was assessed at least 3 months after treatment.

### Statistical and survival analysis

Continuous variables were compared using independent sample t-test and the normally distributed data were expressed as mean ± standard deviation (SD). Comparisons between categorical variables were performed using Pearson’s χ2 test or Fisher’s exact test wherever appropriate and were expressed as n (proportion).

Clinicopathological parameters were assessed by univariate and multivariate Cox proportional hazards regression analysis to identify the independent prognostic factors to RFS and OS. The parameters which showed statistical significance in the univariate Cox regression analysis (*P* < 0.10) were included into the multivariate Cox analysis. The Wald test was used to calculate *P* values. The Kaplan-Meier (KM) estimator and log-rank test was performed to calculate the median survival time and the rates of survival (RFS and OS) and to determine the *P* value.

IBM SPSS 22.0 software was used for the statistical analysis. *P* values (2-tailed) less than 0.05 were regarded as statistically significant.

## Results

### Demographic and clinicopathological characteristics of the study patients

**Fig. 1:**
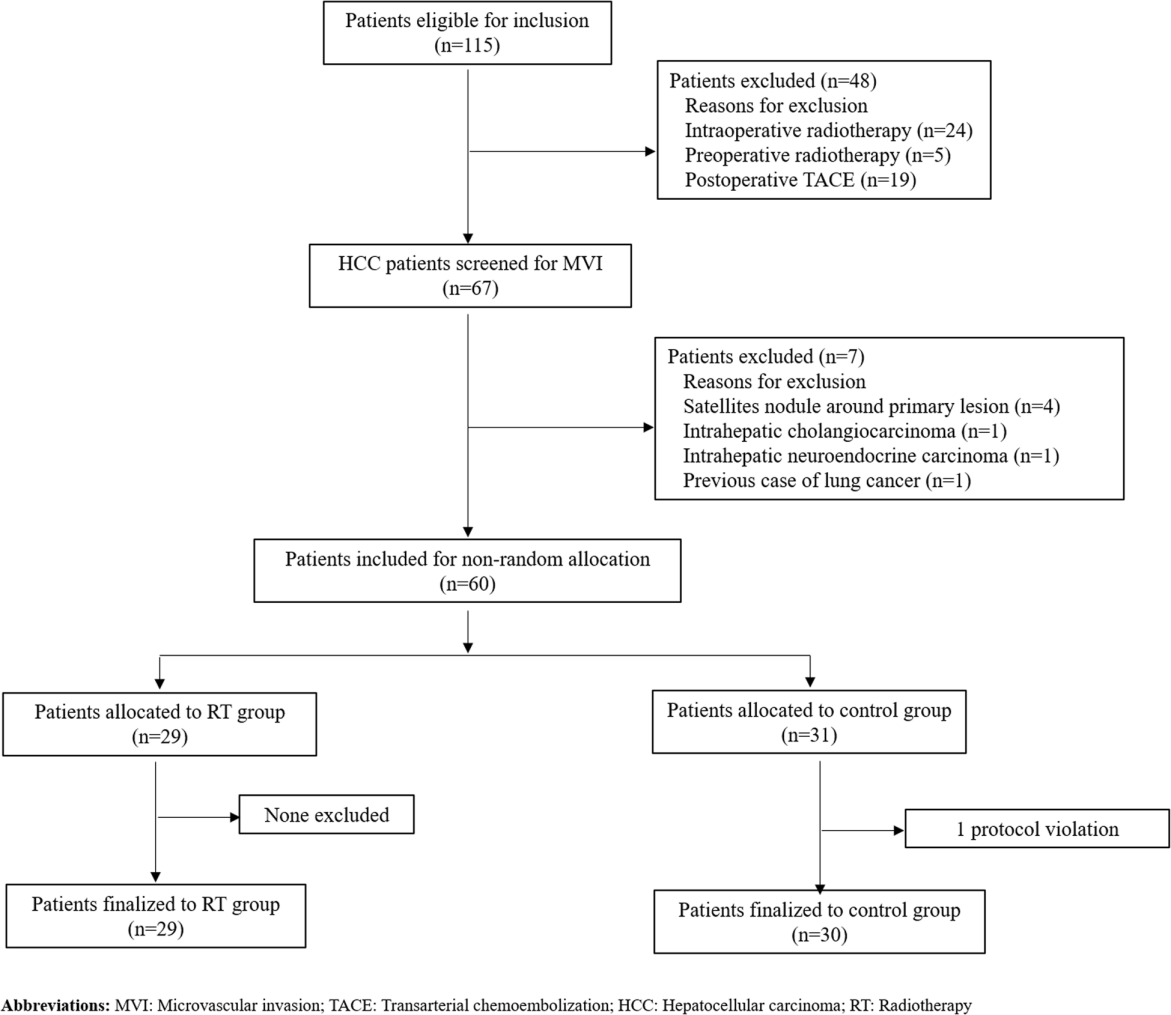
Flow diagram for the study selection of patients eligible for the study

One hundred and fifteen consecutive patients who underwent hepatectomy by the same team met the inclusion criteria for HCC with MVI. Forty-eight patients were excluded from the study since they were treated on other intra or post-operative adjuvant therapy (such as preoperative radiotherapy, intra-postoperative radiotherapy, post-operative TACE, traditional Chinese medicine, etc.). The remaining 67 patients underwent screening for HCC with MVI during which 8 patients were excluded due to various reasons such as: presence of satellite nodule around primary lesions (*n* = 4), intrahepatic cholangiocarcinoma (*n* = 1), intrahepatic neuroendocrine carcinoma (n = 1), previous case of lung cancer (n = 1) protocol violation (n = 1). A total of 59 patients (49 male patients and 10 female patients) with a mean age of 56.24 ± 8.71 (range: 35–72) years old were included in the analysis (RT group, *n* = 29; Control group, *n* = 30). A detailed flowchart for study selection of patients is shown in Fig. 1. The tumor growth pattern of all 59 patients enrolled were nodular. The baseline demographic and the clinicopathological characteristics of the two groups of patients are summarized and compared in Table 1. The baseline characteristics between the two groups were similar and comparable. There were no significant differences between the two groups including age, gender, operative time, blood loss, operative procedure, operative method, surgical margin, tumor size, number of tumor, differentiation, MVI classification, envelope invasion, cirrhosis (fibrosis Scheuer S score), viral hepatitis (HBV-Ag), preoperative serum AFP, alanine aminotransferase (ALT), total bilirubin (TBIL), albumin (ALB) and prothrombin time (PTa).

**Table 1:** Comparisons of Baseline Demographics and Clinicopathological Characteristics between the RT and Control groups

Characteristics	RT (n = 29)	Control (*n* = 30)	*p* value
Age (year)	55.90 ± 8.05	56.57 ± 9.43	0.771
Gender (Male/Female)	24/5	25/5	1.000
Operative time (min)	229.17 ± 75.77	224.00 ± 78.12	0.797
Blood loss (ml)	384.48 ± 317.38	372.67 ± 246.49	0.873
Operative procedure (Major/Minor)	9/20	7/23	0.506
Operative method (Anatomical/Non-anatomical)	14/15	12/18	0.522
Surgical margin (< 0.5 cm / ≥0.5 cm)	19/10	16/14	0.341
Tumor size (cm)	4.75 ± 2.15	4.50 ± 2.98	0.712
Number of tumor (single/multiple)	27/2	28/2	1.000
Differentiation (Well-Moderate/Poorly)	17/12	12/18	0.153
Tumor growth pattern (nodular / diffuse)	29/0	30/0	–
MVI classification (M1/M2)	19/10	21/9	0.713
Envelope invasion (Present/Absent)	14/15	13/17	0.703
Fibrosis Scheuer S score (< 3 /≥3)	14/15	12/18	0.522
HBV-Ag (Negative/Positive)	5/24	3/27	0.417
Preoperative AFP level (Negative/≦400 ng/L / > 400 ng/)	6/16/7	7/16/7	0.970
Preoperative ALT level (U/L)	30.55 ± 18.79	32.67 ± 20.78	0.684
Preoperative TBIL level (umol/L)	13.40 ± 5.32	15.49 ± 6.66	0.188
Preoperative ALB level (g/L)	44.45 ± 3.55	42.72 ± 4.54	0.111
Preoperative PTa level (%)	83.79 ± 11.08	83.81 ± 10.93	0.996

Variables are expressed as the mean ± SD (median with range) or no. (%) (number with percentages), unless otherwise indicated;

*AFP* alpha-fetoprotein; *ALT* alanine aminotransferase; *TBIL* total bilirubin; *ALB* albumin; *PTa* prothrombin time

### Univariate and multivariate analysis for independent prognostic factors

According to univariate analysis, the factors associated with worse RFS were MVI classification (*P* = 0.005) and postoperative treatment strategies (radiotherapy or control) (*P* = 0.009); while tumor size (*P* = 0.056), number of tumors (*P* = 0.095) and envelope invasion (*P* = 0.061) were included in multivariate Cox analysis as their *P* value was > 0.10. Tumor size (*P* = 0.011), MVI classification (*P* = 0.005), postoperative treatment strategies (*P* = 0.015) were identified as factors that influenced worst OS. The multivariate Cox proportional hazard regression analysis revealed that number of tumors (HR = 3.241, 95% CI: 1.077–9.751, *P* = 0.036), MVI classification (HR = 3.539, 95% CI: 1.631–7.681, *P* = 0.001) and postoperative treatment strategies (HR = 0.286, 95% CI: 0.125–0.651, *P* = 0.003) were the independent prognostic factors associated with RFS; tumor size (HR = 1.357, 95% CI: 1.105–1.667, *P* = 0.004), MVI classification (HR = 4.519, 95% CI: 1.460–13.993, *P* = 0.009) and postoperative treatment strategies (HR = 0.094, 95% CI: 0.022–0.397, *P* = 0.001) were the independent prognostic factors associated with OS (Table 2).

**Table 2:** Univariate and multivariate survival analysis of RFS and OS in HCC patients with MVI in the study

Variable	COX			
	Univariate analysis		Multivariate analysis	
	HR (95%CI)	***p*** value	HR (95%CI)	***p*** value
RFS				
Age	0.983 (0.940–1.028)	0.457		
Gender	1.624 (0.487–5.416)	0.430		
Operative procedure	1.038 (0.438–2.458)	0.933		
Operative method	1.116 (0.523–2.382)	0.776		
Operative time	0.998 (0.988–1.009)	0.722		
Blood loss	1.000 (0.999–1.002)	0.673		
Surgical margin	1.325 (0.617–2.843)	0.471		
Tumor size	1.171 (0.996–1.378)	0.056		
Number of tumors	2.490 (0.854–7.261)	0.095	3.241 (1.077–9.751)	0.036
Differentiation	1.750 (0.800–3.825)	0.161		
MVI classification	3.011 (1.396–6.494)	0.005	3.539 (1.631–7.681)	0.001
Envelope invasion	2.118 (0.967–4.638)	0.061		
Fibrosis S score	1.259 (0.582–2.724)	0.558		
HBV-Ag	1.320 (0.397–4.390)	0.651		
AFP level	1.544 (0.900–2.650)	0.115		
Postoperative treatment strategies	0.337 (0.150–0.759)	0.009	0.286 (0.125–0.651)	0.003
OS				
Age	1.011 (0.950–1.077)	0.724		
Gender	0.639 (0.178–2.294)	0.492		
Operative procedure	1.676 (0.561–5.007)	0.355		
Operative method	0.936 (0.325–2.700)	0.903		
Operative time	1.002 (0.989–1.016)	0.743		
Blood loss	1.001 (0.999–1.003)	0.312		
Surgical margin	1.914 (0.663–5.526)	0.230		
Tumor size	1.267 (1.056–1.519)	0.011	1.357 (1.105–1.667)	0.004
Number of tumors	2.354 (0.524–10.567)	0.264		
Differentiation	1.224 (0.424–3.534)	0.709		
MVI classification	4.801 (1.601–14.398)	0.005	4.519 (1.460–13.993)	0.009
Envelope invasion	1.911 (0.639–5.718)	0.247		
Fibrosis S score	2.873 (0.800–10.312)	0.106		
HBV-Ag	1.987 (0.260–15.202)	0.508		
AFP level	1.650 (0.769–3.540)	0.199		
Postoperative treatment strategies	0.204 (0.056–0.737)	0.015	0.094 (0.022–0.397)	0.001

*HR* hazard ratio; *CI* confidence interval

### Survival analysis of the study patients

The cumulative 1-, 2-, 3-year RFS rates of all 59 patients were 66.1, 53.3 and 49.7%, respectively while the cumulative 1-, 2-, 3-year OS rates of all 59 patients were 88.1, 74.3 and 65.7%, respectively.

**Fig. 2:**
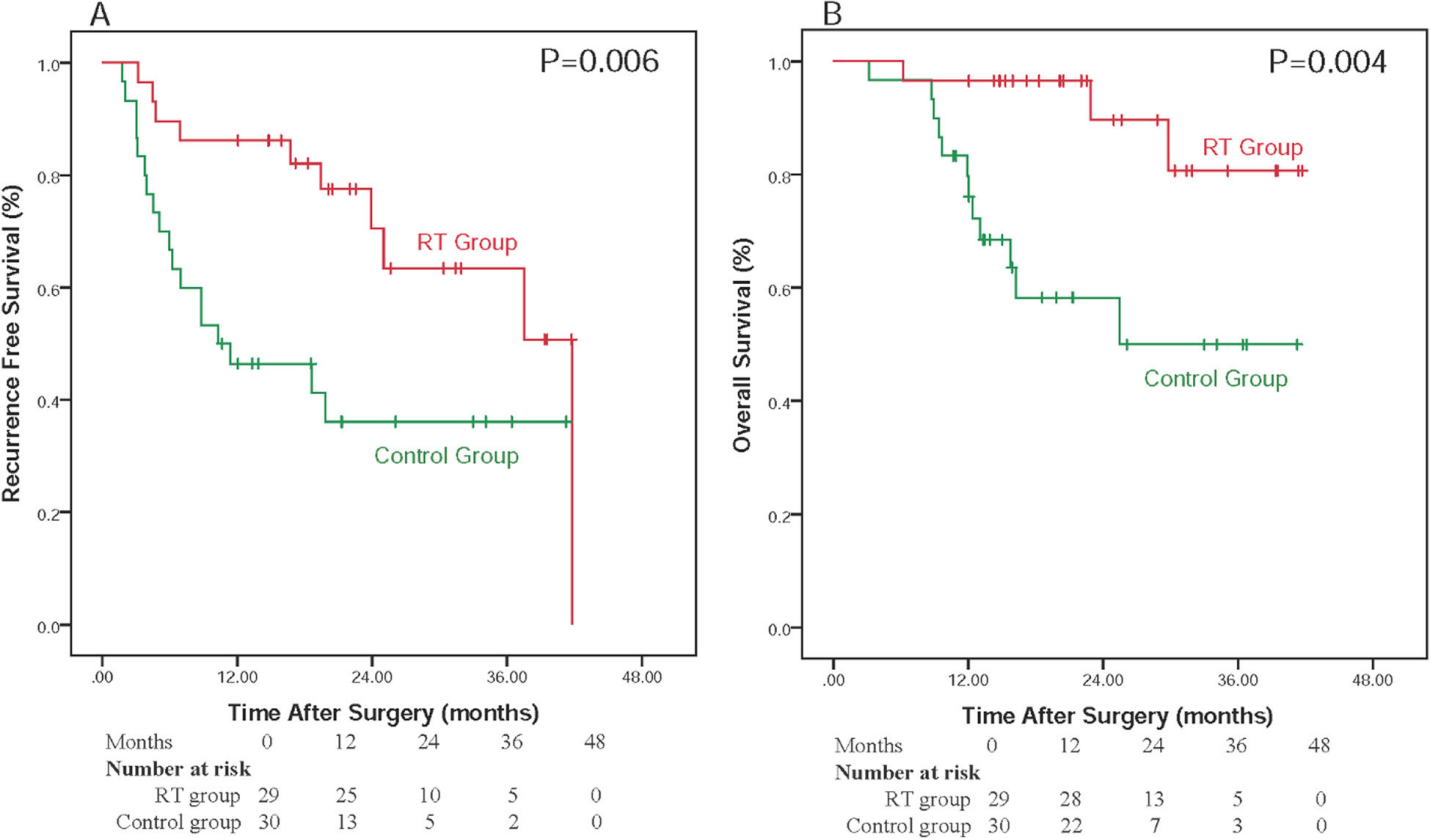
Relapse-free survival and overall survival curves in RT and Control groups

The median duration of RFS for the RT versus Control group was 41.77 versus 10.26 months respectively. The 1-, 2-, 3-year RFS rates were 86.2, 70.5 and 63.4% for patients in the RT group, and 46.4, 36.1, and 36.1% for patients in the control group, respectively. RT group showed a significantly longer RFS rate than the control group (*P* = 0.006). Since relapse within 2 years generally was considered as true recurrence or early recurrence, we calculated the cumulative recurrence rate. The cumulative recurrence rate at 2 year was 29.5% in the RT group and 63.9% in the control group, RT group showed a significantly lower incidence of early recurrence than control group. Median duration of OS for the RT versus control group were 38.11 and 25.44 months respectively. The 1-, 2-, and 3-year OS rates were 96.6, 80.7, and 80.7% for patients in the RT group, and 79.7, 58.3, and 50.0% for patients in the control group, respectively. RT group showed a significantly longer OS than the control group (*P* = 0.004). All of the above data are presented in Fig. 2.

### Survival analysis according to MVI classification

**Fig. 3:**
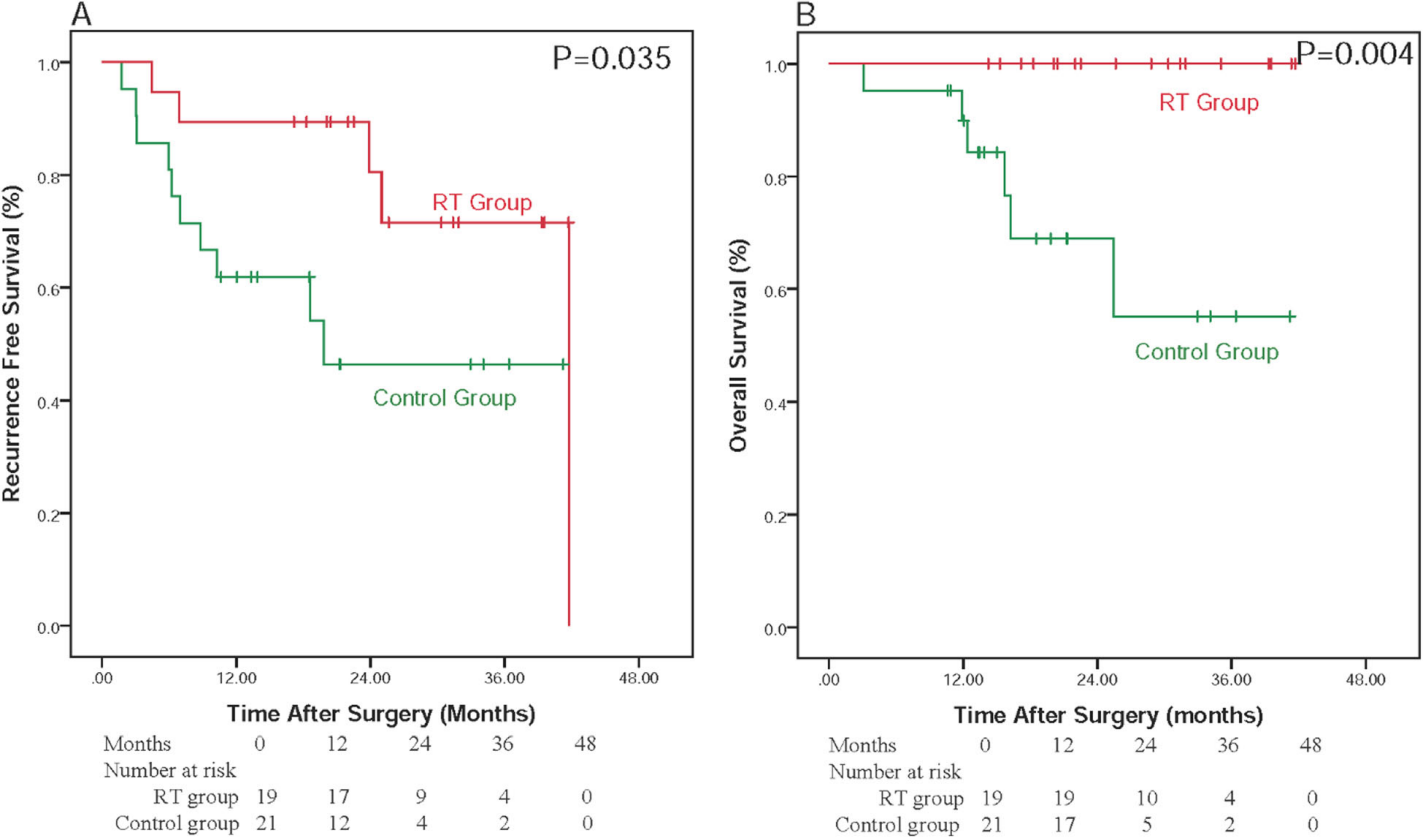
Relapse-free survival and overall survival curves of patients stratified by low grade risk of MVI (M1)

**Fig. 4:**
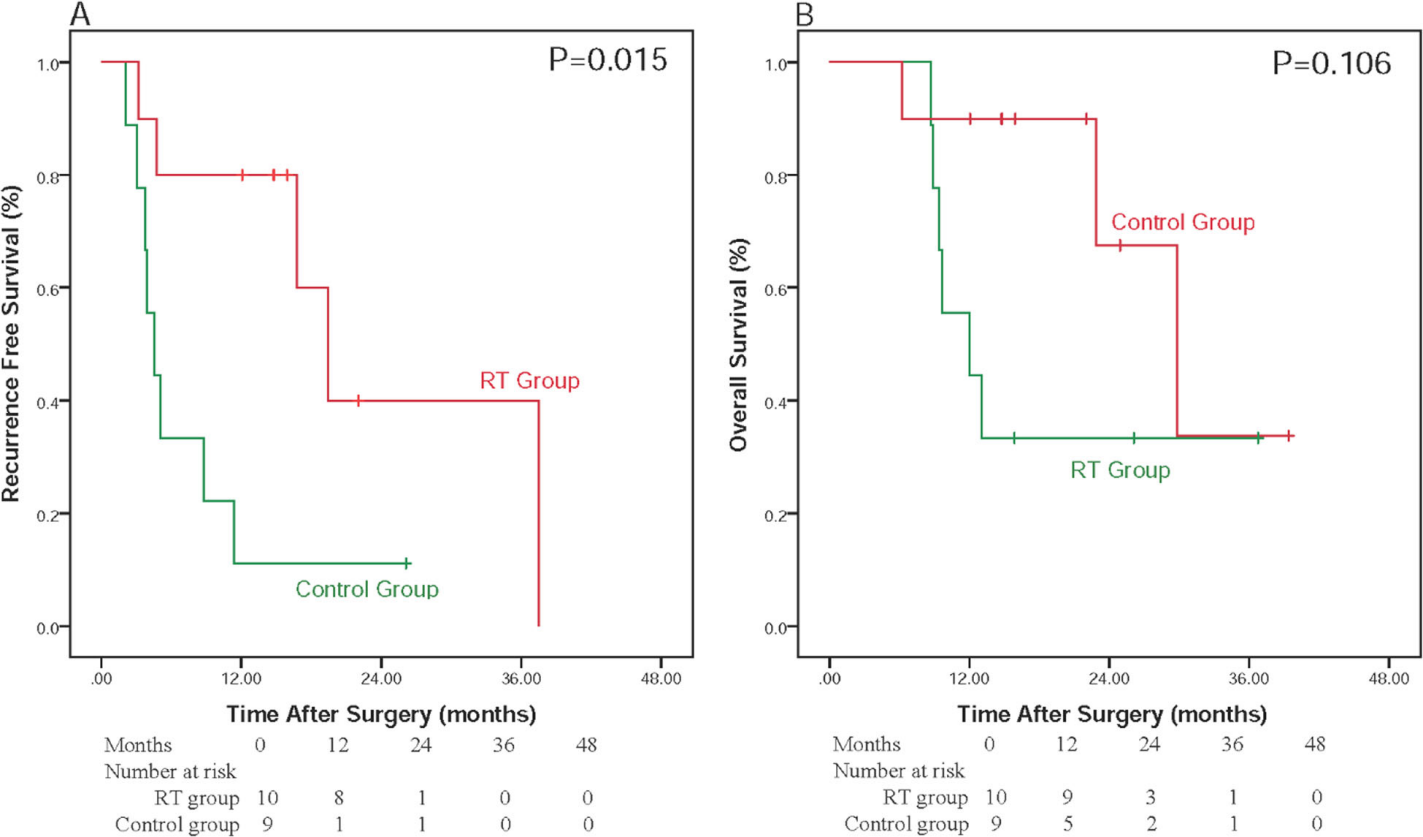
Relapse-free survival and overall survival curves of patients stratified by high grade risk of MVI (M2)

As MVI classification was identified as an independent prognostic factor associated with RFS and OS, the RFS and OS rates were also analyzed in the subgroups of MVI classification. Forty patients were included in the low-risk (M1) microvascular invasion group where the 1-, 2-, 3-year RFS were 89.5, 80.5 and 71.6%, in RT group while was 61.9, 46.4 and 46.4%, in Control group, respectively. The 1, 2 and 3-year OS rates were 100.0, 100.0 and 100.0% in the RT group and 89.9, 69.0 and 55.2% in the control group, respectively. RT group showed a significantly longer RFS (*P* = 0.035) and OS (*P* = 0.004) than the control group (Fig. 3). Nineteen patients were included in the high-risk (M2) MVI group, where the 1-, 2-, 3-year RFS rates were 80.0, 40.0 and 40.0%, in RT group and 11.1, 11.1 and 11.1% in control group, respectively. The 1-, 2-, 3-year OS rates were 90.0, 67.5 and 33.8% in RT group, 55.6, 33.3 and 33.3%% in the control group, respectively. The median duration of RFS and OS was found to be 19.44 and 29.80 months in RT group while 4.56 and 12.03 months in control group, respectively. RT group showed a significantly better RFS (*P* = 0.015) than control group (Fig. 4a), however, though RT group depicted a longer OS than control group, the results were not significant (log rank *P* = 0.106) (Fig. 4b).

### Recurrence pattern in two groups

Recurrence was observed in 28 of 59 patients, with 10 patients and 18 patients in RT and control group, respectively. The incidence of intrahepatic and extrahepatic recurrence was 8 and 2 in RT group, 13 and 5 in control group, respectively, with no significant difference between the two groups (*P* = 1.000). For patients with intrahepatic recurrence, the incidence of marginal and non-marginal recurrence was 3 and 5 in RT group, 2 and 11 in control group, respectively, and no significant difference was observed between the two groups (*P* = 0.325); the incidence of nodular and diffuse recurrence was 6 and 2 in RT group, 6 and 7 in control group, respectively, with no significant difference between the two groups (*P* = 0.367). For patients with extrahepatic recurrence, the incidence of limited and disseminated recurrence was 2 and 0 in RT group, 1 and 4 in control group, respectively (*P* = 0.143). The details of the pattern of recurrence is shown in Table 3.

**Table 3:** Pattern of recurrence and treatment in the RT and Control groups

Recurrence Pattern	RT	Control	*p* value
Location (for all)	10	18	1.000
intrahepatic	8 (80%)	13 (72.2%)	
extrahepatic	2 (20%)	5 (27.8%)	
Location (intrahepatic)	8	13	0.325
Margin	3 (37.5)	2 (15.4%)	
non-margin	5 (62.5)	11 (84.6)	
Growth Pattern (intrahepatic)	8	13	0.367
Nodular	6 (75%)	6 (46.2)	
diffuse	2 (25%)	7 (53.8)	
Growth Pattern (extrahepatic)	2	5	0.143
limited	2 (100.0%)	1 (20%)	
disseminated	0 (0%)	4 (80.0%)	

### Toxicity of adjuvant radiotherapy

Toxicities usually were observed in the acute stage, while late stage toxicity was rare. Five patients (7.8%) experienced grade 1 or 2 nausea, 3 patients (3.9%) complained of grade 1 or 2 anorexia, 4 patients experienced grade 1 fatigue, 6 patients experienced grade 2 gastritis or duodenitis while 5 patients experience grade 1 dermatitis. Myeloid suppression (*n* = 21) and liver dysfunction (*n* = 13) was the most common toxic effect; however, only 3 (10.34%) patients developed grade 3 myeloid suppression. All these patients recovered from the acute toxicities after 1–3 weeks of treatment, and no patient had any interruption of irradiation. No grade 4 toxicity was observed. Moreover, no patient had developed radiation-induced liver disease, increase in creatinine, gastroduodenal ulcer and other severe late toxicities. Vomiting and diarrhea were seldom observed. The observed irradiation-related toxicities were mild. The toxicities associated with RT are summarized in Table 4.

**Table 4:** Radiotherapy-related toxicities in patients who underwent Postoperative Radiotherapy

	Number of Patients			
Toxicity	Grade 0	Grade 1	Grade 2	Grade 3
Nausea	24	4	1	0
Vomiting	27	2	0	0
Anorexia	26	2	1	0
Gastroduodenal ulcer	29	0	0	0
Gastritis or duodenitis	23	6	0	0
Diarrhea	28	1	0	0
Respiratory infections	0	1	0	0
Fatigue	25	4	0	0
Dermatitis	24	5	0	0
Myeloid suppression	8	12	6	3
Liver dysfunction	16	13	0	0
Creatinine increasing	29	0	0	0

## Discussion

Curative hepatectomy is considered as a standard treatment modality for HCC patients with preserved liver function. Unfortunately, because of the high incidence of recurrence, the long-term survival after hepatectomy is not satisfactory. MVI has been reported to be one of the most important negative factor associated with recurrence and significantly poor RFS and OS following curative resection [20, 33–35]. In a previous study, MVI increased the odds of recurrence (Odds Ratio (OR): 28.40) and decreased survival (OR:4.70, 95% CI: 1.24–17.80) [33]. In patients with HCC who had undergone liver transplantation, analysis in patients with high MVI showed significantly poorer outcomes than other groups for RFS (*P* = 0.003) [36]. In another study, the 5 year OS for patients beyond the Milan criteria and with MVI was 27.27% whereas for patients beyond the Milan criteria and without MVI was 57.89% (*P* = 0.003) [37]. Similarly, in the patients with or without MVI, the 1-year RFS was 12% vs. 69% while the 3-year OS rate was 16% vs. 58%, respectively [38]. A meta-analysis of 20 studies was carried out to address the prognostic impact of MVI and found that patients with MVI had significantly reduced disease-free survival and OS at 3- and 5-years after liver resection and transplantation [9]. This was in accordance with our study where both the univariate and multivariate analysis showed that the MVI classification was one of the major prognostic factor for depiction of worst RFS and OS in HCC patients.

MVI disseminate mainly via portal venous branches and spread along as well as against the direction of the portal venous flow, [39] thus it is regarded as the anatomic prerequisite for tumor spread in circulation [13]. The presence of MVI is associated with multiple factors such as tumor size, morphology and degree of differentiation of hepatic neoplasms [33, 40, 41]. A study reported that the incidence of MVI was almost twice as high in tumors larger than 5 cm (61%) as in smaller tumors (32%). A study reported that invasion of a vessel with a muscular wall, invasion of a vessel ≥1 cm from the tumor capsule and invasion of > 5 vessels were significantly associated with recurrence [16]. Determination of anatomic resection with the optimal margin has been widely examined for its effect on postoperative outcome in patients with HCC, however its significance is controversial across various studies. A resection margin of 2 cm is effective and safe in decreasing the postoperative recurrence rate and improving survival outcomes when compared to resection margins between 1 cm and ≤ 2 cm [39]. Few studies proposed a resection margin of at least 1.0 cm to eradicate microscopic lesions for the majority of patients to reduce recurrence [42, 43]. Few studies reported that a wider resection margin is preferable to eradicate microscopic lesions [42, 43]. However, most of patients in these cohorts displayed cirrhosis to certain extent. A very important consideration for patients with cirrhosis is to preserve non-tumorous liver parenchyma as much as possible to prevent postoperative liver failure. This also improves the chances of performing multimodality treatment and repeat resections in case of tumor recurrence. For this purpose, a narrow margin should be considered as a better choice for HCC patients [44–47]. In our study a narrow margin of 1-cm was preferred for all the patients to administer RT. This resulted in fewer incidences of recurrences in RT group than in control group.

There is no established adjuvant therapy to prevent the recurrence of HCC. Various postoperative adjuvant therapies for HCC after curative resection such as TACE, RT, molecular targeted therapies and immunological therapies have been evaluated as strategies to reduce recurrence and thus prolong OS. However, the outcomes of these interventions are variable [16]. RT had been traditionally avoided for treatment in HCC because of the risk of radiation induced liver disease and limited response. However, advances in technology has led to the development of advance external RT techniques, such as three-dimensional conformal or intensity-modulated RTs, which delivers tumoricidal radiation doses precisely to tumor bed area and spares the normal liver tissues, without incurring significant radiation. Recent clinical studies demonstrated that postoperative RT provided better survival outcomes in HCC patients. A systematic review of 24 studies with 4349 HCC patients with MVI showed that the median OS decreased from approximately 50% at 1 year to 18% at 5 years, while the median DFS decreased from 32 to 18% from 1-year to 5 years [48]. Wang et al. investigated the benefit of postoperative intensity-modulated radiotherapy (IMRT) in patients receiving narrow margin hepatectomy for HCC located close to major vessels and found that IMRT improved 3-year overall and disease-free survival without severe liver damage [26]. Additionally, the effect of adjuvant radiotherapy in patients who underwent narrow margin hepatectomy for centrally located HCC < 5 cm found significant difference between the RT and control groups in the OS and RFS at 3- and 5-years [30]. In our previous retrospective studies [21, 22] postoperative adjuvant RT significantly improved RFS and OS compared to TACE and conservative therapy which implies postoperative adjuvant RT, could eliminate residual micrometastasis foci in the remnant liver. In the present study, patients who underwent curative hepatectomy alone had significantly shorter RFS and OS than in patients who underwent curative hepatectomy plus postoperative adjuvant RT.

A correlation between higher MVI grade and shorter disease-specific survival and RFS has been noted [17]. MVI has been associated with two main prognostic significance: the invasion of vessels ≥1 cm from the tumor capsule and the number of invaded vessels ≥5 [16]. A study by Roayaie et al. observed that invasion of a vessel with muscular wall, invasion of a vessel ≥1 cm from tumor capsule and invasion of > 5 MVIs were significantly related to postoperative recurrence and were also predictors of survival [16]. In our study, univariate and multivariate analysis showed that MVI classification was an independent factor for both RFS and OS. We conducted a subgroup analysis according to MVI classification. Survival analysis demonstrated that postoperative RT resulted in significantly superior survival outcomes than in control group regardless of the degree of MVI classification. Also, no significant difference in the OS between the groups of patients with M2 was observed. The likely explanation for this insignificance may be due to the limited number of cases. Based on our data, we deduced that postoperative RT might control persistent residual microscopic lesions in the remnant liver tissue in either M1 or M2 patients.

There was no significant difference in the recurrence pattern between the two groups. Given the fact that the RT group had significantly better RFS and lower early recurrence rate than the control group, postoperative radiotherapy could reduce both intrahepatic and extrahepatic recurrence. In other words, the survival in the RT group prolonged due to the reduction in both intrahepatic and extrahepatic recurrence. We speculate the following reasons for these results. A few studies had found that occult microscopic lesions such as MVI may still reside in the remnant liver tissue surrounding HCC after hepatectomy. The potential remaining microscopic lesions can either cause local recurrence in situ (margin) or spread through the portal and hepatic veins, causing non-margin or even extrahepatic recurrence. Our data revealed that postoperative radiotherapy could reduce the probability of early intrahepatic recurrence, both marginal and diffuse, and extrahepatic recurrence.

To our knowledge, this is the first study in Chinese patients to investigate the efficacy of postoperative adjuvant RT in HCC patients with MVI following curative hepatectomy against the standard postoperative therapy and subsequently determine the prognostic factors for recurrence in HCC patients with MVI. However, there are few limitations that warrant mention. The postoperative RT therapy was not randomized, which may introduce bias between the groups, although the patients’ baseline was similar among the two groups. Also, due to relatively small sample size, further stratification analysis could not be carried out. However, the results from our study do provide rationale for developing a randomized clinical study with larger sample.

## Conclusions

Postoperative adjuvant RT following hepatectomy found better outcomes with RFS for HCC patients with MVI than with standard postoperative therapy. Thus, postoperative RT will be useful to control the microscopic lesions in remnant liver tissue in both M1 (low risk) and M2 (high risk) subgroups of HCC patients with MVI.

## Data Availability

The datasets used and/or analysed during the current study are available from the corresponding author on reasonable request.

## Abbreviations

MVI: Microvascular Invasion
HCC: Hepatocellular Carcinoma
HBV: Hepatitis B virus
HCV: Hepatitis C virus
OS: Overall Survival
RFS: Relapse free survival
RT: Radiotherapy
LR: Liver resection
TACE: Transcatheter arterial chemoembolization
SBRT: Stereotactic body radiation therapy
IMRT: Intensity modulated radiotherapy
PASS: Power analysis and sample size
CT: Computed Tomography
MRI: Magnetic resonance imaging
RFA: Radiofrequency ablation
ALP: Alkaline phosphatase
SD: Standard Deviation
ALT: Alanine aminotransferase
TBIL: Total bilirubin
ALB: Albumin
PTa: Prothrombin Time
